# Identifying site- and stimulation-specific TMS-evoked EEG potentials using a quantitative cosine similarity metric

**DOI:** 10.1371/journal.pone.0216185

**Published:** 2020-01-13

**Authors:** Michael Freedberg, Jack A. Reeves, Sara J. Hussain, Kareem A. Zaghloul, Eric M. Wassermann

**Affiliations:** 1 Behavioral Neurology Unit, National Institute of Neurological Disorders and Stroke, Bethesda, MD, United States of America; 2 Henry M. Jackson Foundation for the Advancement of Military Medicine, Bethesda, MD, United States of America; 3 Human Cortical Physiology and Neurorehabilitation Section, National Institute of Neurological Disorders and Stroke, Bethesda, MD, United States of America; 4 Functional and Restorative Neurosurgery Unit, National Institute of Neurological Disorders and Stroke, Bethesda, MD, United States of America; Universita degli Studi di Trento, ITALY

## Abstract

The ability to interpret transcranial magnetic stimulation (TMS)-evoked electroencephalography (EEG) potentials (TEPs) is limited by artifacts, such as auditory evoked responses produced by discharge of the TMS coil. TEPs generated from direct cortical stimulation should vary in their topographical activity pattern according to stimulation site and differ from responses to sham stimulation. Responses that do not show these effects are likely to be artifactual. In 20 healthy volunteers, we delivered active and sham TMS to the right prefrontal, left primary motor, and left posterior parietal cortex and compared the waveform similarity of TEPs between stimulation sites and active and sham TMS using a cosine similarity-based analysis method. We identified epochs after the stimulus when the spatial pattern of TMS-evoked activation showed greater than random similarity between stimulation sites and sham vs. active TMS, indicating the presence of a dominant artifact. To do this, we binarized the derivatives of the TEPs recorded from 30 EEG channels and calculated cosine similarity between conditions at each time point with millisecond resolution. Only TEP components occurring before approximately 80 ms differed across stimulation sites and between active and sham, indicating site and condition-specific responses. We therefore conclude that, in the absence of noise masking or other measures to decrease neural artifact, TEP components before about 80 ms can be safely interpreted as stimulation location-specific responses to TMS, but components beyond this latency should be interpreted with caution due to high similarity in their topographical activity pattern.

## Introduction

Transcranial magnetic stimulation (TMS)-evoked EEG potentials (TEPs) are a rich source of neurophysiological data and have been used to study a variety of phenomena, including consciousness [1,2], memory [3], and the pathophysiology of neurological disorders [4–6].

Recent work has demonstrated that TEPs differ in waveform shape across recording and stimulation sites [7], and are affected by pharmacological interventions [8] and brain states [7,9], reflecting their cortical origin. However, a major factor limiting the interpretation of TEPs as cortical responses to TMS is the presence of time-locked artifacts. The influence of these artifacts on the recorded TEP remains a matter of significant debate [10,11]. Artifacts from sources outside of the brain, such as electrode noise and recharge artifacts from the TMS coil, do not interact directly with brain signals and can be removed offline with techniques such as independent component analysis (ICA) [12,13]. However, artifacts from cortical responses to auditory or somatosensory stimulation produced by the TMS pulse can interact with, and even modify, the amplitude of neural signals [14,15]. Therefore, sensory artifacts cannot be isolated and removed reliably with ICA [16] or by subtracting the amplitude of sham-evoked auditory and somatosensory artifacts from active TMS responses. Although sensory artifacts can be attenuated with noise masking and vibration-damping of the coil [1,17], recent work [18] has indicated that even when data are collected under tightly-controlled conditions, resulting TEPs may still be contaminated by artifact.

Due to the likely impossibility of completely removing brain-generated artifacts, such as auditory and somatosensory responses, it is important to identify which epochs of the TEP contain such artifacts and to interpret signals from those epochs with caution or avoid them altogether. Previous studies have shown that a late TEP component, the N100, is similar in scalp distribution irrespective of stimulation site and between active and sham stimulation [19,20]. The lack of spatial selectivity suggests that the N100 does not result from direct cortical activation by TMS [7,20] and that it and later components are affected by time-locked sensory artifact or some other non-specific process [17,21,22]. A study using noise masking and reduction of sensation with foam material between the coil and scalp [20] supported this theory by showing significant correlations between active and sham-evoked waveforms near N100 latencies. Correlated waveforms indicate the presence of waveform components that are common across stimulation conditions (active and sham) in amplitude-independent timing and patterns of evoked activity. Correlation analysis is preferable to amplitude-based methods, which only compare the voltage at a single electrode between conditions [20] and can lead to misidentification of waveforms as local brain responses due to ill-controlled or incompletely removed artifact. However, correlation analysis is limited by lower time resolution than amplitude-based methods.

Alternatively, by calculating the instantaneous cosine similarity between waveforms, similarity can be assessed at each sampled time point. Cosine similarity compares evoked responses across multiple channels and can be used to measure similarity in the shapes of responses across a set of electrodes [23]. In order to use it, the waveform is transformed to a binarized, amplitude-independent series of values representing the direction of change at each successive timepoint after the pulse. The binarized value for each timepoint from a set of waveforms can be represented as a vector and the cosine of the angular difference between any two vectors provides a measure of waveform similarity at that timepoint [24]. In this way, similarity in TEPs can be assessed by comparing binarized waveforms from different conditions (i.e. between-condition similarity). Additionally, by comparing a set of TEPs within the same condition, cosine similarity can be used to determine the repeatability of responses (i.e. within-condition repeatability). Although this approach has limitations (see Discussion), it has been used successfully to detect similarity in neurophysiological signals [23].

We used the cosine similarity method to measure similarity in TEPs resulting from active and sham TMS delivered to three different stimulation sites, with the hypothesis that TEPs generated by stimulation at different sites should produce different topographic distributions of activity when the recording is not dominated by non-site-specific activity, i.e., artifact. The objective of this study was to demonstrate the ability of the cosine similarity method to detect artifact.

## Methods

### Participants

Twenty healthy individuals (11 F; mean age 24.3 ± 3.1 years) provided informed consent to participate in the study, which was approved by the NIH Combined Central Nervous System Institutional Review Board. All experiments were performed in accordance with the Declaration of Helsinki.

### MRI acquisition and TMS targeting

MPRAGE T_1_-weighted scans were obtained from each participant using either a GE 3T HDx scanner (N = 16, 32-channel head coil, voxel size = 1 cm^3^, field of view = 25.6 cm^2^, flip angle = 7°, 172 transverse slices) or a Siemens 3T Skyra scanner (N = 4, 32-channel head coil, voxel size = 0.926 cm x 0.926 cm x 0.900 cm, field of view = 23.7 cm^2^, flip angle = 9°, 192 sagittal slices). Predetermined TMS target locations for the right dorsolateral prefrontal cortex (DLPFC; MNI: 42 46 4) [25] and left posterior parietal area (PPC; MNI -48–65 45) [26] were prepared for each participant by transforming their anatomical scans to MNI space, placing a 3 mm radius circular marker at the target location, and transforming the scans back to subject space using AFNI neuroimaging analysis software [27]. The target location for the primary motor area (M1) was determined empirically (see “TMS and EEG recording” section). For PPC stimulation, the handle of the coil was oriented approximately 10° laterally from the antero-posterior axis [26]. For DLPFC stimulation, the handle of the coil was oriented 45° medially from the anterior-to-posterior axis. For M1 stimulation, the coil angle was adjusted to the orientation that maximized the motor response, which was typically 45° relative to the mid-sagital line [28] (see “TMS and EEG recording” section).

### TMS, EMG, and EEG recording

A Brainsight frameless neuronavigation system was used to guide stimulation to the left PPC, left M1, and right DLPFC targets in each participant. Monophasic TMS was delivered with a 70 mm figure-of-eight coil connected to a Magstim 200 stimulator. Electromyographic responses were recorded from the right first dorsal interosseous muscle with bipolar surface electrodes arranged in a belly-tendon montage. The target for M1 stimulation was the scalp site at which stimulation required the lowest intensity to evoke at least five motor-evoked potentials (MEPs) > 50 uV on ten consecutive trials. Resting motor threshold (RMT) was calculated using an automatic threshold-tracking algorithm (TMS Motor Threshold Assessment Tool, MTAT 2.0) [29]. Stimulation intensity for active and sham stimulation at all sites was set to each participant’s RMT, which was, on average, 45.9 ± 9.37% (mean ± SD) of maximum stimulator output.

### Experimental design and recording

We delivered 150 active and 50 sham TMS pulses at each stimulation site. The order of sites was counterbalanced between participants. Pulses were delivered every five seconds with a 15% jitter to reduce any effects of pulse anticipation. EEG signals were sampled at 5000 Hz (DC filtered at 1000 Hz) from 30 recording scalp electrodes in a 10–20 arrangement using a TMS-compatible EEG system (BrainAmp MR+, Brain Vision). Electrode impedances were maintained at < 10 kΩ. The goal of sham stimulation was to reduce cortical stimulation without eliminating the auditory or somatosensory artifacts. This was achieved by placing a 3 cm cardboard spacer between the coil and scalp. For each location, active TMS was performed first, followed by sham. Participants had foam earplugs placed during all TMS procedures.

### Data preprocessing

**Fig 1:**
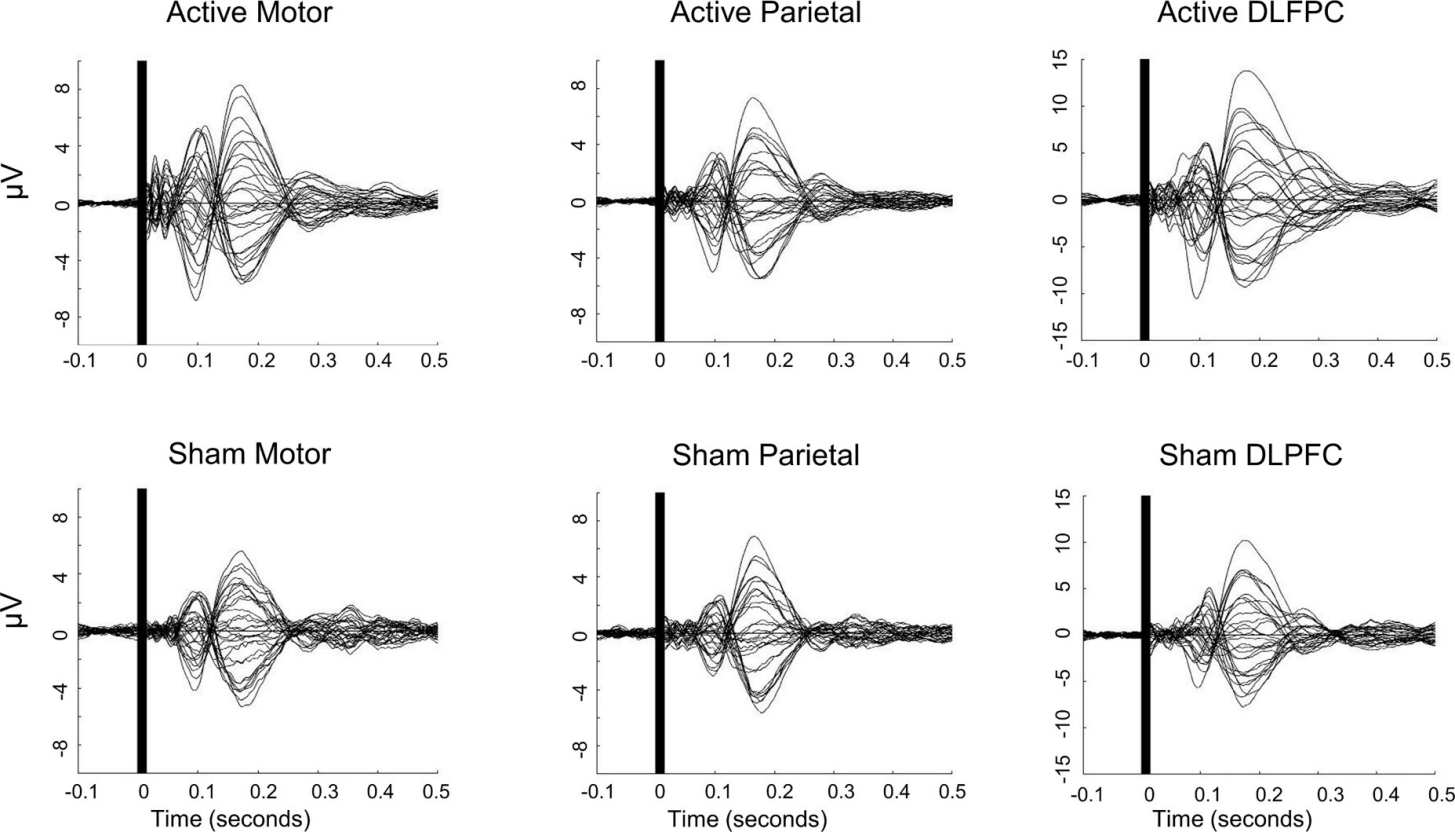
Group-averaged EEG voltage traces for active and sham M1, PPC, and DLPFC stimulation. Plots show average voltage traces for EEG channels FP1, FP2, F3, F4, C3, C4, P3, P4, O1, O2, F7, F8, T7, T8, P7, P8, FZ, CZ, PZ, IZ, FC1, FC2, CP1, CP2, FC5, FC6, CP5, CP6, TP9, and TP10.

**Fig 2:**
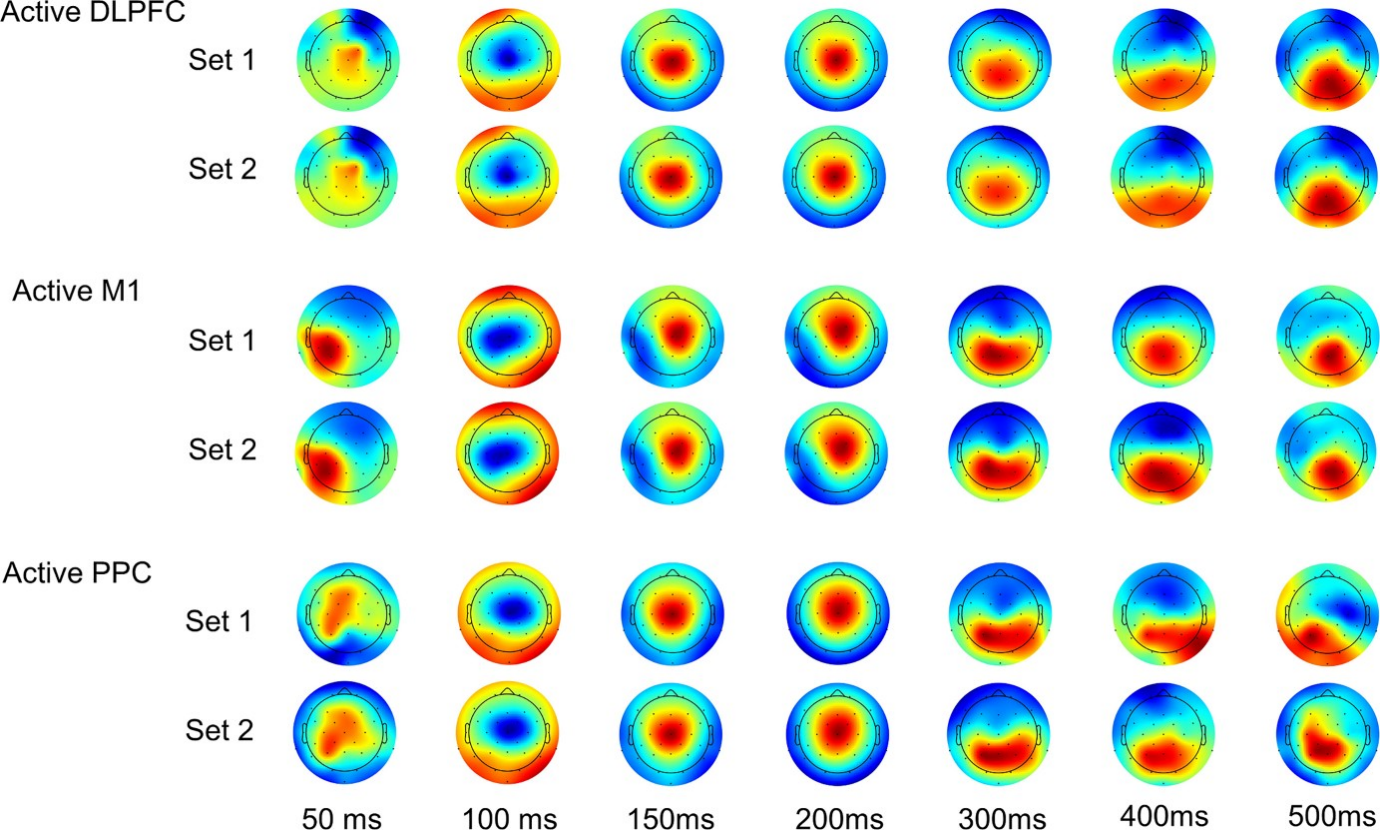
Group-averaged topographical distributions for active stimulation. Plots are shown for every 50 ms from 50 to 500 ms. Each set of plots is the average from a set of 75 randomly selected trials from each subject.

We preprocessed the data with a standard analysis pipeline (TMS-EEG Signal Analyzer (TESA) extension for EEGLAB [12] running in a MATLAB environment). Trials were epoched from -1.5 to +3 sec around each TMS pulse and data from -1 ms to +15 ms around each pulse were removed and replaced with a cubic interpolation. The data were down-sampled to 1000 Hz and noisy channels were automatically detected and removed if they exceeded a full-epoch kurtosis measurement of 5. On average, we removed one channel per participant for each stimulation condition (minimum = 0; maximum = 3). We baseline-corrected each epoch by whole-epoch de-meaning and then performed two rounds of ICA to detect and remove artifacts. Both rounds used the FastICA algorithm [30] with a symmetric decomposition approach and hyperbolic tangent contrast function. The first round of ICA included an automated artifact detection algorithm (tesa_compselect) [12] to remove large pulse-related artifacts. The second round was performed to eliminate small artifacts not rejected during the first round. To obtain a consensus about which components should be rejected, authors JR, SH, and MF performed agreement training on a test data set. Each author individually performed components rejection on subsets of test data until substantial inter-rater agreement was achieved (> 0.78 Cohen’s kappa interrater coefficient) [31,32]. Author JR performed the final component rejection for the second round of ICA. Between the two rounds of ICA, data were processed with a 1–100 Hz zero-phase bandpass Butterworth filter and a 58–62 Hz zero-phase band-stop filter to remove line noise. Following ICA, we baseline-corrected individual trials from -100 ms to -10 ms relative to TMS pulse. Channels were re-referenced to the average of all channels for each trial. Finally, we calculated the time-locked average waveforms for each participant and each condition and visually inspected each participant’s preprocessed TEPs by plotting the trial-average for each EEG channel recorded at each stimulation site. We performed an additional data quality check by inspecting plots of group-level channel averages (Fig 1). Group-level averages were also created to show the topographical distribution of voltages in each active stimulation condition (Fig 2). These were split into two sets of 75 random trials for each subject and averaged across subjects to show the consistency of the group-level responses.

### Similarity analysis

We used a modified cosine similarity method [23] to measure between-condition similarity and within-condition repeatability in evoked topographical activity patterns. Prior to analysis, we Hjorth-transformed [33] our data to reduce the influence of volume conduction. We used a within-subjects design to compare evoked responses for each participant separately, with statistically significant differences calculated at the group level (see “Statistical analyses of similarity measurements,” below). To identify patterns of similarity, we compared TEPs between the active to sham stimulation conditions at each stimulation site and active to active stimulation between stimulation sites, resulting in six sets of between-condition similarity measurements: three between-site similarity curves (M1-Parietal, M1-DLPFC, DLPFC-Parietal), and three between-stimulation type similarity curves (M1 active-M1 sham, Parietal active-Parietal Sham, DLPFC active-DLPC sham). We also computed the within-condition repeatability of responses to active stimulation at each site by splitting the trials obtained for each active stimulation condition into two non-overlapping samples and comparing them using the cosine method. The within-condition repeatability analysis provided a practical maximum level of similarity for that site to contrast with the similarity in the between-condition and location comparisons. This accounted for noise and other factors that decreased the maximum similarity level from the theoretical value of one.

**Fig 3:**
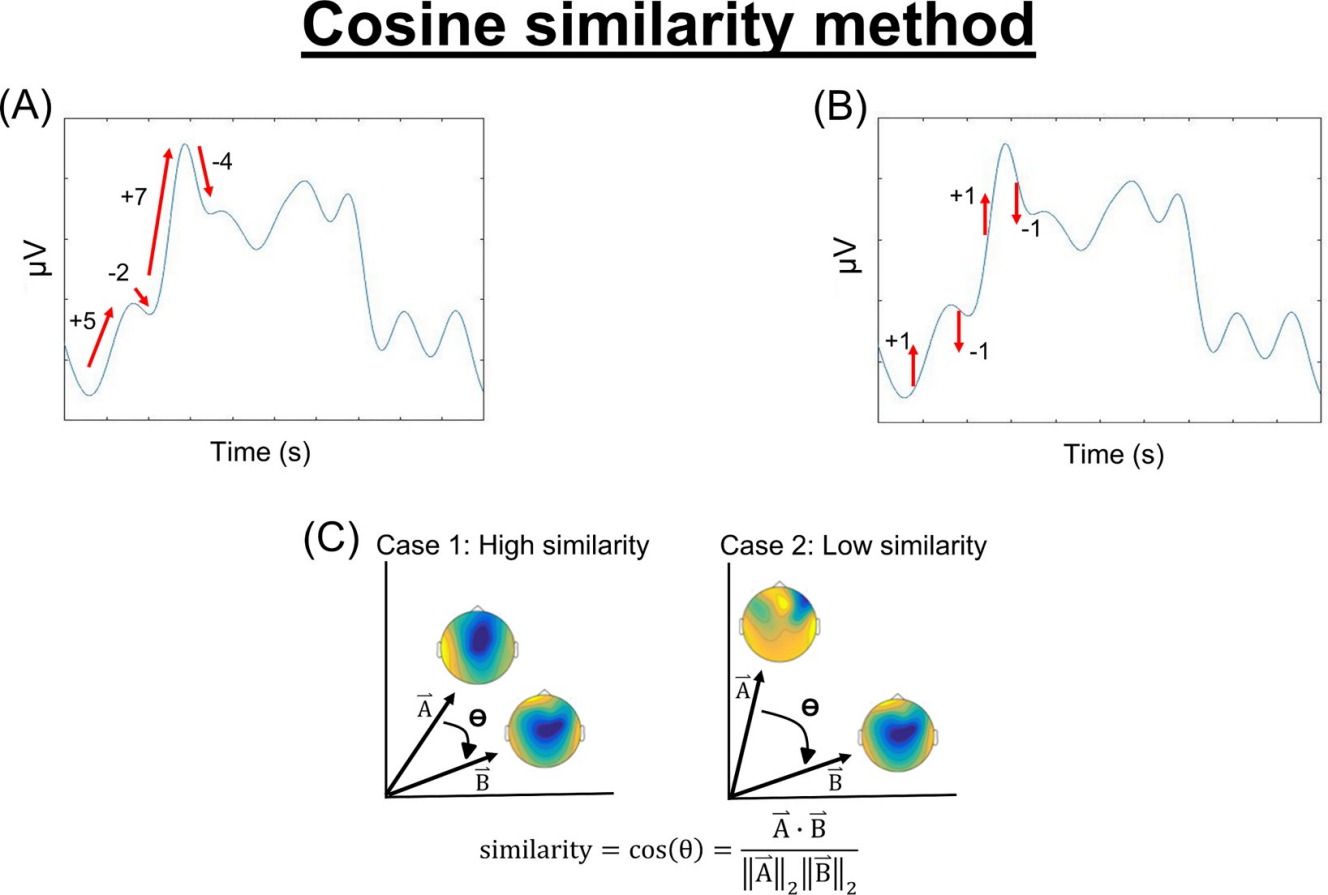
For all stimulation conditions, we took the derivative of the preprocessed and averaged TEPs at every time point for each channel (A), binarized the derivatives so that an increase in point-to-point amplitude was assigned a value of +1 and a decrease a value of -1 (B), and calculated cosine similarity between the two “feature vectors” (C).

For each condition, we created an average waveform from 50 randomly-selected trials from each participant. Since only 50 sham trials were delivered, we used 50 trials to equalize the number of trials between comparisons (active vs. sham and between-site active stimulation). Non-overlapping trials were used for within-condition repeatability comparisons. We obtained the derivatives of the preprocessed and averaged TEPs at every time point for each channel by subtracting the amplitude of each timepoint from the amplitude of preceding one (Fig 3A). The derivatives were then binarized so that an increase in point-to-point amplitude was assigned a value of +1 and a decrease a value of -1 (Fig 3B). This removed the amplitude dimension of the data and allowed for model-free analysis of the shape of the waveform. The binarized values were input into a “feature vector” [23], where each EEG channel corresponded to a certain vector index. For each comparison, cosine similarity between the two feature vectors was calculated (Fig 3C) at each time-point using all channels as follows:

$$S_{t}=\frac{\sum_{i=1}^{n}A_{i,t}B_{i,t}}{\sqrt{\sum_{i=1}^{n}{\left(A_{i,t}\right)}^{2}}\sqrt{\sum_{i=1}^{n}{\left(B_{i,t}\right)}^{2}}}$$ (1)

where *S*_*t*_ is cosine similarity at time *t*, *A_i,t_* and *B_i,t_* are the binarized derivatives for each condition at channel *i* and time *t*, and *n* is the total number of channels. The process was repeated 1000 times for each participant and each comparison, using randomly-permuted trial averages. The within-participant mean of the averages was then calculated as the measure of similarity between stimulation sites (between-site similarity), stimulation types (between-stimulation similarity) and within-condition repeatability.

### Statistical analyses of similarity measurements

For the between-condition similarity and within-condition repeatability analyses (see above), we used cluster-based permutation testing [34] to perform group-level comparisons. Four types of tests were conducted: 1) comparisons of all similarity curves to pre-stimulus baseline values, 2) comparisons of responses within each stimulation condition (within-condition repeatability), 3) comparisons of similarity between sites (between-site similarity curves), and 4) comparisons between active and sham stimulation similarity at each site (between-stimulation similarity curves).

We performed statistical comparisons to reveal significant differences between baseline and post-TMS within-condition repeatability and between-condition similarity. For each comparison, each participant’s average within-condition repeatability and between-condition similarity curves were segmented into baseline (1500–500 ms before TMS) and response (15–1015 ms after TMS) epochs. A *p-*value was calculated for each time point by comparing the distributions of the participants’ baseline and response epochs. Clusters of significant time-points were identified by summing the *p-*values of all contiguous time points that reached a threshold of 0.05. We controlled for multiple comparisons by comparing each of the cluster sizes to a distribution of 1000 *p-*value clusters derived from randomly permuted epoch comparisons and retaining only original clusters > 95% of the random cluster distribution. Each random cluster was created by comparing two randomly-sampled distributions of twenty epochs each from the combined pool of the baseline and response epochs and selecting the size of the largest cluster by summed *p-*value, as above.

## Results

### Group-level TEPs

Active TMS produced significant cortical activation at the M1, PPC, and DLPFC sites, with maximum group-level peak-to-peak amplitudes across the scalp of 13.9 μV, 12.9 μV, and 23.0 μV, respectively (Fig 1). Sham TMS also produced significant activation at all three sites but with lower amplitude than active stimulation (M1: 10.9 μV, PPC: 12.5 μV, DLPFC: 17.9 μV).

**Fig 4:**
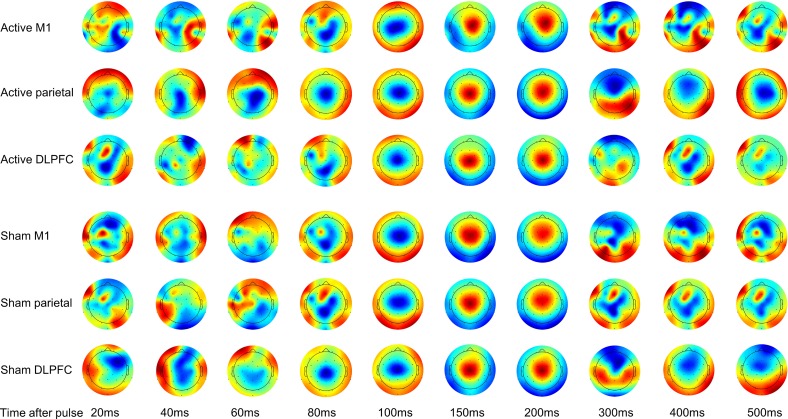
Group-level topographic activity plots for each stimulation condition. Plots are scaled to the minimum and maximum electrode voltage at that timepoint to emphasize spatial activation patterns consistent with the amplitude-independent nature of the cosine similarity approach.

Qualitatively, TEPs differed spatially between 15 ms (end of interpolation) and 60 ms (Fig 4). Between 60 and 80 ms, spatial patterns began to converge across conditions. By 100 ms, the responses were highly similar and remained so until approximately 300 ms.

### Within-condition repeatability of topographical patterns of activation

**Fig 5:**
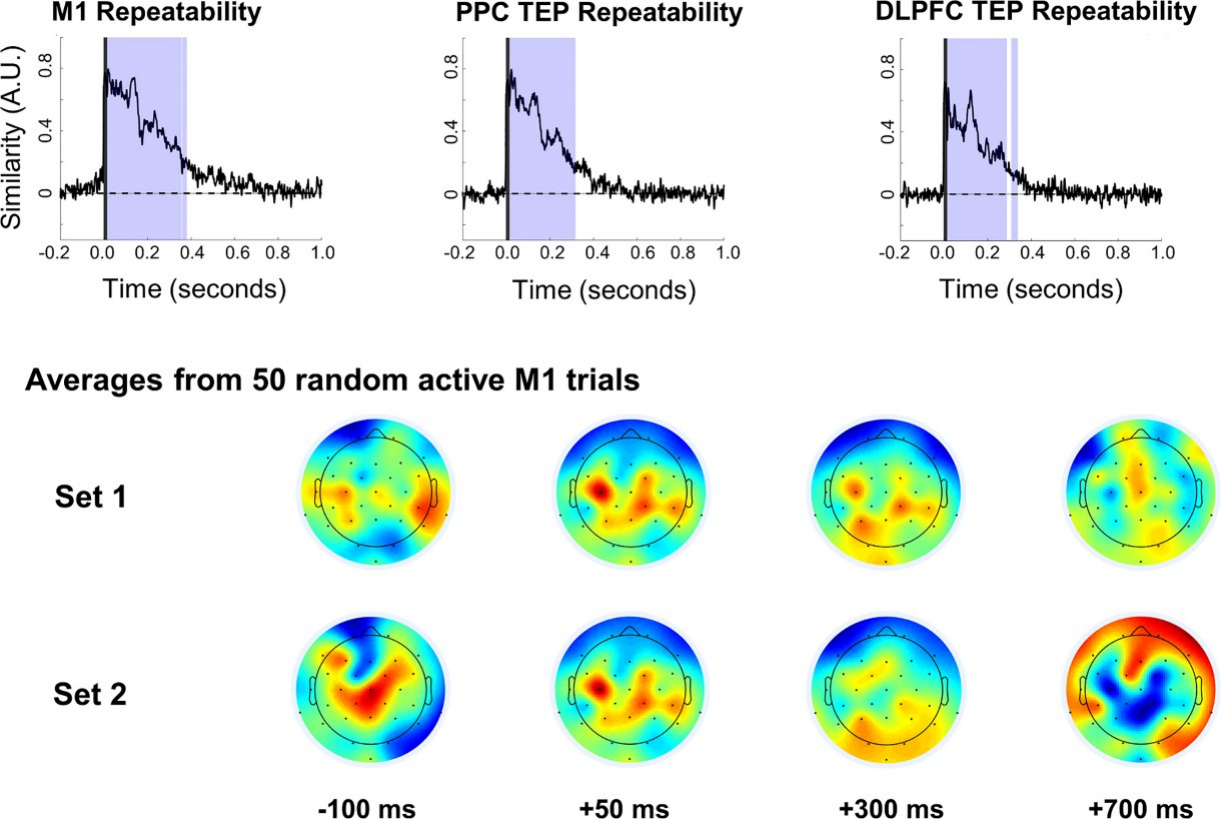
Top: Group-level within-condition repeatability curves comparing within-participant and within-condition averages for active stimulation. The y-axis indicates arbitrary similarity units with a possible range of -1 to +1. Light Shading shows periods of significant difference from pre-stimulation baseline. The dark shaded regions from -1 ms to 15 ms indicate TMS pulse interpolation. Bottom: Plots show topographical distributions of two averages from active M1 stimulation, constructed using 50 non-overlapping trials each from one participant.

The within-condition repeatability of TEP waveforms was maximal immediately following the pulse for all stimulation sites and decayed to pre-stimulus values by 346 ± 26 ms after the TMS pulse (Fig 5). Significant differences from baseline started at 15 ms (the end of interpolation) and extended to 381 ms for M1, 318 ms for PPC, and 339 ms for DLPFC active stimulation. A large drop in within-condition repeatability occurred at a mean of 136 ms after TMS for all three locations.

### Between-site and between-stimulation similarity

**Fig 6:**
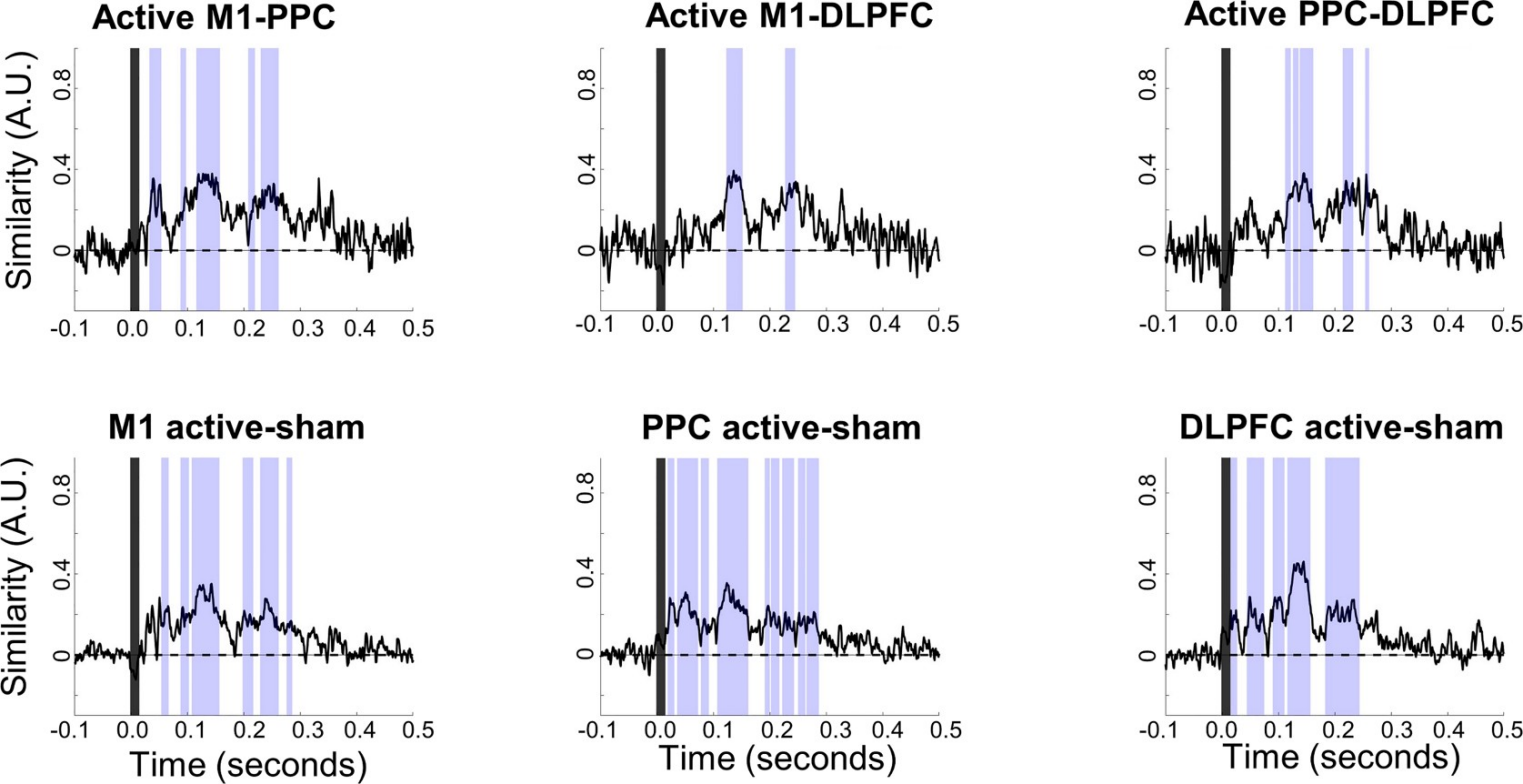
Between-site and between-condition similarity curves. Lightly shaded regions shows epochs of significant difference from baseline, indicating that similarity between the compared conditions is above chance. The darkly shaded regions from -1 ms to 15 ms indicate TMS pulse interpolation.

Two epochs of significantly increased similarity from baseline were common to all six between-site and between-stimulation comparisons (Fig 6). The first was between 126 ms and 152 ms, and the second was between 230 ms and 233 ms, after the TMS pulse. Increases in similarity before these epochs started at 90 ± 14 ms and 196 ± 13 ms. Significant between-condition similarity was present prior to 80 ms after the TMS pulse in all but the active M1-DLPFC and DLPFC-PPC comparisons. However, unlike the later two epochs, no < 80 ms epochs were consistently identified across all comparisons, indicating site-specific patterns of activity, rather than stereotyped signal from artifact. Before 80 ms, an average of two, and a maximum of four, comparisons overlapped in their epochs of significant similarity. Additionally, the onset of the initial epoch of significance differed between active-to-active comparisons (approximately 30 ms post-pulse) and between active-to-sham comparisons (approximately 89 ms post pulse). This similarity in the topographical pattern of activation suggests the presence of artifact under active and sham stimulation conditions at the same stimulation site, but not between stimulation locations. Overall, similarity between all conditions decayed to baseline values by 263 ± 18 ms (Fig 6).

## Discussion

We compared similarity in the spatial distributions of TEPs from active and sham TMS, from active stimulation at different sites, and between samples of trials from the same sites using the cosine similarity method. These comparisons enabled identification of epochs during which TEP activation patterns were similar between sites and conditions, indicating the presence of neural artifact, likely due to auditory and/or somatosensory activation. Our results are consistent with previous findings [18], in that the waveforms of early responses (15–80 ms after TMS) under different stimulation conditions were significantly correlated (Fig 6). However, the specific epochs with statistically significant similarity differed between comparisons. For example, TEPs from active M1 TMS were similar to active PPC TEPs from 30–54 ms, while active M1 TEPs differed from active DLPFC TEPs until 112 ms after TMS. This indicates that the events in the period from 15–80 ms did not contain an artifact common to all stimulation conditions and are consistent with claims [20,21] that TEP components 15–80 ms after the TMS pulse are condition and site specific. We note, however, that somatosensory effects are likely to vary by stimulation site and cannot be distinguished from signal caused by direct cortical stimulation. Across all comparisons, there was a rapid change from low to high similarity at 90 ± 14 ms, corresponding with the onset of the N100 component. Our findings are consistent with the consensus that N100 is a nonspecific response [18,19,35,36].

We found that the within-condition repeatability of whole-brain TEP patterns decreased over time (Fig 5), with a large drop at approximately 136 ms after TMS, indicating that the latencies of TEP components occurring before 136 ms can be precisely and reliably estimated in our data, while later latencies may require higher number of trials to estimate with similar precision. While the origin of this drop in repeatability is not clear, the overall result is consistent with a previous study [37], which showed higher between-session repeatability in peak amplitude and latency for early components (≤ 100 ms) than for a late component corresponding to the P180. However, our results differ with those of a later study [38] which found P180 amplitude to be equally repeatable as the early N40 and P60 components for within-session comparisons. A possible reason for this discrepancy is that the latter study [38] analyzed a subset of electrodes which was biased toward the vertex and prone to sensory contamination [35], which could artificially increase repeatability. It is important to note that these studies used amplitude-dependent repeatability measures. Our cosine-based within-condition repeatability results quantify consistency in both temporal and spatial distribiutions of responses to TMS, factors which cannot be explicitly accounted for in amplitude-based studies.

The objective of this study was to use cosine similarity to distinguish between responses to cortical stimulation and neurally generated sensory artifacts, such as auditory and somatosensory responses, in the TEP. Therefore, we did not attempt to mask these stimuli. We acknowledge that our results may apply only when auditory and somatosensory artifact is present, although Conde et al. [18] found undifferentiated signal from 70–200 ms which is consistent with our results. We also note that cosine similarity analysis is compatible with noise masking, and that combining the two should help improve the ability to detect artifacts occurring during noise masking. Our data may also be helpful for future studies where noise masking is not applicable or appropriate. For example, the presence of background noise can differentially affect task performance across experimental groups, such as children with ADHD versus healthy controls [39], and introverts versus extroverts [40], and can also affect performance on cognitive and perceptual tasks [41].

The cosine similarity approach as applied here has limitations. First, it is based on temporal similarity across EEG channels. Non-source localized EEG data suffers from poor spatial resolution due to volume conduction. We chose not to source-localize our data because we only used 30 active channels, which is likely insufficient for solving the inverse problem [42]. However, applying the Hjorth transformation to our data did not affect our results, suggesting that our findings are not likely caused by volume conduction. Second, it is possible that stimulating different cortical sites can activate the same networks, particularly at longer latencies [43]. Therefore, any observed between-condition similarity could feasibly be due to either sensory artifact or stimulation at different cortical sites converging to evoke similar networks over time.

## Conclusions

Cosine similarity is a simple and effective tool for identifying TEP epochs when neural artifact is likely to be present. Although artifact may be present throughout the TEP response, our results show that early components best reflect site and condition-specific responses to cortical stimulation. In contrast, later responses become progressively more similar between stimulation conditions, suggesting that these components predominately reflect neural artifacts. We suggest that only TEP components occurring prior to 80 ms are safely interpretable as condition and site-specific responses to cortical stimulation when noise masking is not used. In addition, TEP waveform reproducibility decays monotonically with increasing time after the TMS pulse. While later components may contain valuable physiological information, we suggest caution in in their interpretation and warn against their attribution to direct cortical stimulation by the TMS pulse. Auditory and somatosensory stimulation controls and/or further refinements in signal processing may be necessary to extract true late responses to cortical stimulation.
